# ChemScanner: extraction and re-use(ability) of chemical information from common scientific documents containing ChemDraw files

**DOI:** 10.1186/s13321-019-0400-5

**Published:** 2019-12-11

**Authors:** An Nguyen, Yu-Chieh Huang, Pierre Tremouilhac, Nicole Jung, Stefan Bräse

**Affiliations:** 1 Institute of Toxicology and Genetics, Karlsruhe Institute of Technology, Hermann-von-Helmholtz-Platz 1, 76344 Eggenstein-Leopoldshafen, Germany; 2 Institute of Organic Chemistry, Karlsruhe Institute of Technology, Fritz-Haber-Weg 6, 76131 Karlsruhe, Germany

**Keywords:** Data mining, Chemical data extraction, CDX, CDXML, Molecule recognition

## Abstract

We developed ChemScanner, a software that can be used for the extraction of chemical information from ChemDraw binary (CDX) or ChemDraw XML-based (CDXML) files and to retrieve the ChemDraw scheme from DOC, DOCX or XML documents. This can facilitate the reuse of chemical information embedded into diverse documents used as standard storage and communication instrument in chemical sciences (e.g. for student’s theses, PhD theses, or publications). The extracted information is processed to reactions, molecules, as well as additional text and values and can be accessed via the ChemScanner UI. ChemScanner supports the export to Excel and CML, the direct import of the extracted data to the Open Source ELN Chemotion or the use via “copy and paste” of selected information. The software was designed with a focus on the processing of documents with embedded molecular structure information as CDX or CDXML as these are the most common file formats for chemical drawings. The project aims to support the chemists in their efforts to re-use chemistry research data by providing them missing tools for an automated assembly of reaction data.

## Background

The re-usability of scientific results is of high importance. It has a high impact on the speed and accuracy of scientific developments and the development of common knowledge. The relevance of the reusability of research data is highlighted by many initiatives and institutions, amongst which are the FAIR (findable, accessible, interoperable, and re-usable) data initiative [1] and the RDA (research data alliance) [2]. As in all other disciplines as well, the re-usability of research data in chemistry depends on manifold factors. Relevant factors are, for example, the availability and the frequent use of repositories, the standardization of scientific documentation and data formats, the use of ontologies as well as metadata, and the availability of software to analyze and process the available data. To the current situation, the re-usability of research data is far from being perfect as repositories are not used on a permanent basis, standardization projects, ontologies, and metadata are upcoming but have not yet reached the whole community, and software to analyze and process the research data is often not available to all researchers. In addition, research in chemistry suffers from one particular and fundamental requirement: chemistry research is in most of the cases connected to chemical structures which have to be presented in a user readable and understandable format. To be able to present the chemical structure in a way that allows the exchange of information, usually embedded ChemDraw drawings are used. ChemDraw from CambridgeSoft (now a product of PerkinElmer) [3] is the de facto standard chemical structure editor in the industry and is also probably the most common editor in academia. The editor allows the application of a broad toolbox of chemical visualization tools and offers many additional functions for the generation of calculated information and identifiers. Although there are some alternatives to ChemDraw, like ISISDraw (MDL Information Systems, now a product of BIOVIA) [4], MarvinSketch (ChemAxon) [5] or Open Source software like Ketcher (EPAM Life Sciences) [6, 7], JChemPaint [8], and JSME [9] which are in particular useful for an application with databases, electronic lab notebooks and diverse web-based applications, ChemDraw is still, to our knowledge, the most commonly used editor. Its use is not only preferred for the generation of reports and for documentation but also for the generation of adequate pictures for publications. For the latter purpose, the documents containing embedded ChemDraw information are processed to a PDF and published together with the supporting information, which is given as PDF as well. This working habit for publishing chemistry results makes a reuse of chemical structures a time-consuming effort. Chemical structures generated with ChemDraw are usually embedded in the proprietary file format CDX or CDXML and cannot be processed easily. If the document including the molecular structures is processed to a PDF document, the situation is even worse, as the information can only be obtained by OCR (optical character recognition) at the moment. Therefore, the re-use and automated capture of molecular information from scientific documents is very often the bottleneck of an automated extraction of chemical information. As the interest of the community on the information captured in scientific documentations is very high, tools to overcome the current situation have been developed during the last years. Extraction tools covering diverse aspects of chemical data extraction have been offered by commercial vendors or have been released as Open Source [14]. A web-tool that allows the processing of names and identifiers from text, PDF or URLs is chemicalize.org [10]. Chemicalize was initiated by ChemAxon that also offers software for the comprehensive data extraction with OCR tools (document to structure [11]). An OCR application for chemical structures (OSR—Optical Structure recognition) can also be obtained as Open Source with OSRA (NIH) [12, 13]. OSRA can read documents with over 90 graphical formats and automatically recognizes and extracts the graphical information as chemical structures which can be obtained as SMILES or SDF output. Additionally, many other helpful tools that support the extraction of textual information have been developed [14]. One example is OPSIN, an Open Source software to convert identified chemical (IUPAC) names into structures [15–18]. OPSIN can be used as a web-service or can be used as a JAVA library for further development. Beneficial applications like the combination with algorithms to automatically detect chemical names in documents (CHEMDNER) [19] or software to analyze chemical experiment language (OSCAR) [20, 21] could be of interest in future. The success and scope of past approaches of document mining were demonstrated several times with patent data, for example by the SureChEMBL project [22] (extraction of 10 million molecules) or the extraction of chemical reactions by Lowe (more than 1 million reactions) [23]. Despite these mostly recent developments, the community does not benefit from readily available tools for the extraction of chemical information from native CDX(ML) files or document-embedded CDX(ML) schemes. Open source software such as the cheminformatic toolkits OpenBabel [24] and RDKit [25] are able to analyze CDX(ML) files and can be used for simple drawings. To the best of our knowledge, open source tools for the extraction and curation of complex molecules, reactions and pathways are missing so far.

## Implementation

ChemScanner was implemented to the software Chemotion ELN core to allow the full compatibility with the functions of an ELN. This offers options like the storage and management of extracted information or the editing and correction of information which are not part of the ChemScanner code. Chemotion-ELN, programmed in Ruby on Rails with ReactJS, is a development of our research group which has been reported earlier [26]. ChemScanner implemented to Chemotion-ELN is available as a web service via the website: https://www.chemotion.net. The source code of ChemScanner is published under the open source license AGPLv3 on GitHub [27]. As ChemScanner is developed as a Ruby gem, it can be used in any kind of Ruby application. The basic use of ChemScanner includes the five classes CDX, CDXML, DOC, DOCX, and XML (from Perkin Elmer’s ELN [28]) corresponding to the possible file types that can be uploaded for the extraction of information. The output of an extraction of information from the uploaded files is a molecule, a reaction, a set of molecules or a set of reactions. Reactions always contain three groups: reactants, reagents, and products. Each group is a set/array of the molecule(s). Each molecule representation can be accessed via ‘cano_smiles’ for Canonical SMILES, and ‘mdl’ for Molfile. Reagents/solvents SMILES which are converted from—abbreviations are also accessible via ‘reagents_smiles’. In addition, other information like the reaction time, temperature, and yield, or the status of the reaction, are also properties of the output. The options export to CDXML and CML are part of the ruby gem ChemScanner, allowing the use of the software for the generation of reaction libraries. ChemScanner uses a customized Ruby version of RDKit [29] as the chemistry toolkit of the development.

## Results

### Selection of file types and overview of the ChemScanner UI

**Fig. 1:**
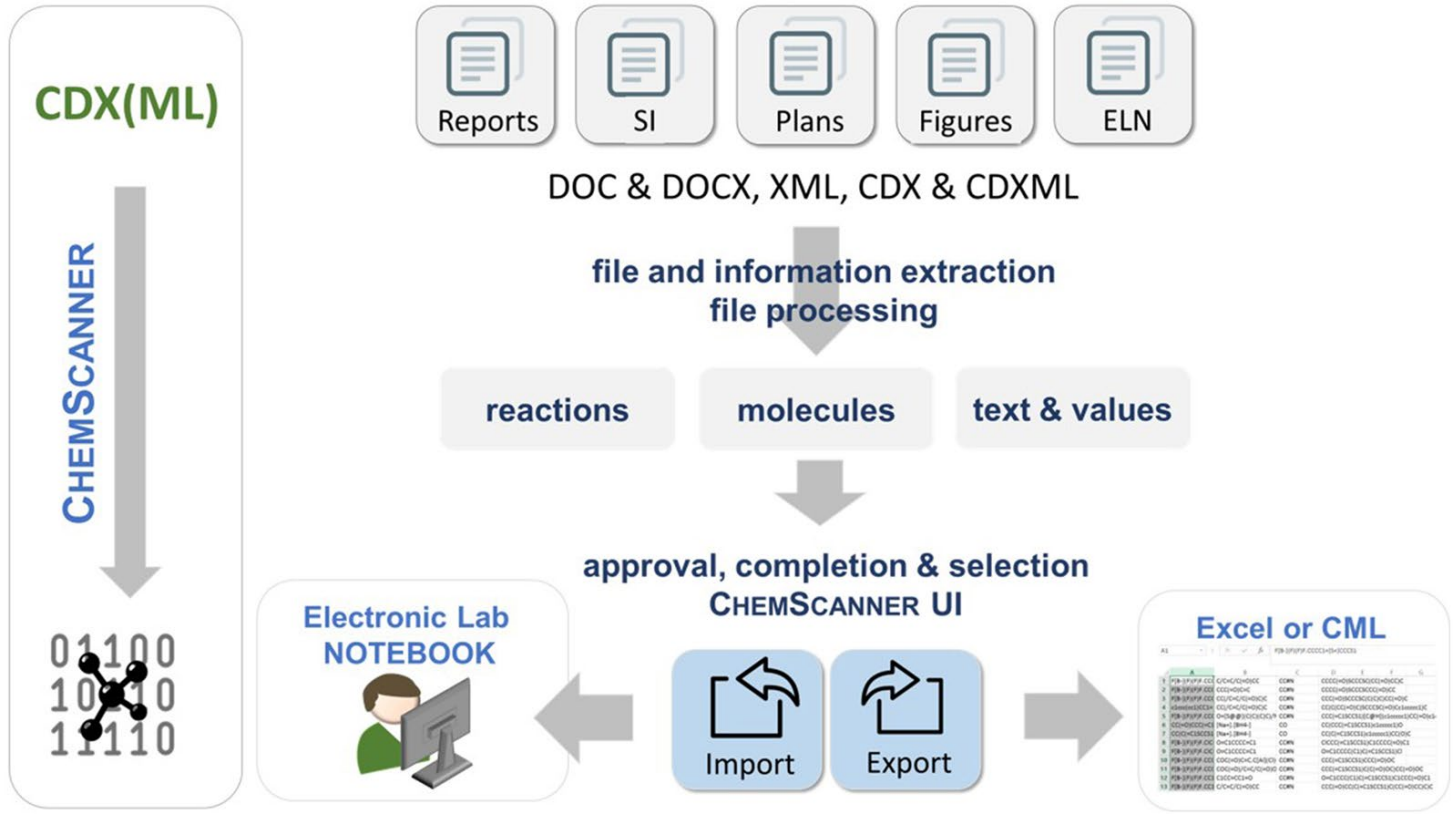
Schematic representation of the core functions of the ChemScanner software [30]

In chemistry documentation and research communication, the most common file types are Word-documents with embedded ChemDraw structures, ChemDraw native files or PDF documents. These formats are used by most of the synthetic chemists and were analyzed at the beginning of our work towards their potential for re-usability. The documents in DOC or DOCX file format serve e.g. as templates for publications and supporting information or as an exchange format for information in report form. Quite common is also the preparation of synthesis plans and structure collections with the ChemDraw editor and the storage of the drawing as native CDX (ChemDraw exchange) file. In addition to the documents used by almost every chemist, we also analyzed options to extract information from XML files that were gained as export file format from the Perkin Elmer ELN. The latter ELN, available through the ChemOffice package, was and is used by many chemists but the export and re-use of reaction data are almost unfeasible. For all of the documents of interest, either the CDX or CDXML file format plays a key role for the successful extraction of information. Solutions to the re-use of this CDX or CDXML data as well as the parsing of documents that contain these file formats are presented with the herein described software. PDF which is a common format used by the publishers, implies the loss of lot of information during the conversion and its retrieval relies on an error-prone processing by OCR (OSR) techniques. The reconstruction of information from PDF is not part of this work as structures are given as images only. We decided to focus on the parsing of documents that still contain structural information on molecules as binary or text content. In its current version ChemScanner can manage DOC, DOCX, CDX, CDXML and XML files. The files can be read by ChemScanner and the available information is extracted and processed to reactions, molecules, and additional text and values. The data can be re-used by exporting it to Excel, to CML or by importing the data directly into the Chemotion ELN as a management system (for an overview of the functions of ChemScanner see Fig. 1). The software was designed with a focus on the processing of molecular structure information in combination with textual data to allow scientists to re-use documents on scientific results in an easy way. ChemScanner in its current version uses only structure and textual information from schemes, other information given in the documents is not extracted.

### Option 1: Processing of CDX and CDXML files

The file formats CDX and CDXML are often used for the capture of chemical information. The binary format CDX is the native file format for ChemDraw and is used most often to write structures in ChemDraw. In addition, the CDX format allows the embedding of chemical structures into the Word files DOC or DOCX while maintaining the consistency and the synchronization of the ChemDraw information. The CDXML file is a CDX file specially formatted so that it conforms to the XML specification. CDXML allows a better compatibility with databases like the SQL server and therefore is also used in the ELN offered by Perkin Elmer (ChemDraw-ELN). The parsing of these two file types CDX and CDXML is of high value as the content can be used to process and retain most of the important information that was generated via the ChemDraw editor. Both file formats contain chemical objects (e.g. atoms, bonds, reactions) and properties (e.g. charge, valence, atom number, bond order) as structure content and in addition text or text fragments that are used to describe additional structure or reaction conditions. The parsing of CDX and CDXML files therefore consists of an analysis with respect to its structure content and additional textual information.

**Fig. 2:**
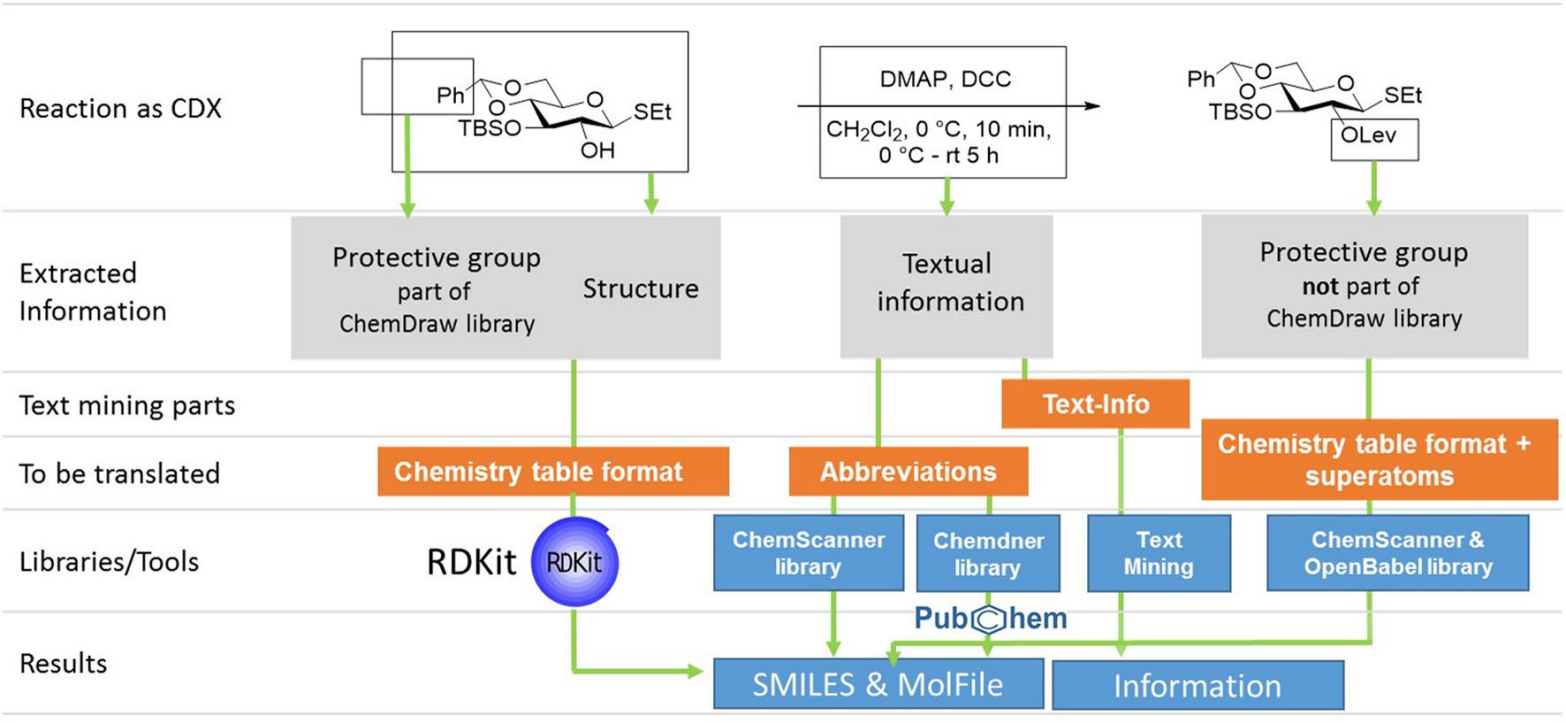
Exemplarily chosen schematic representation of the procedure to extract and process different information from CDX files

Structure-related information can be retrieved from CDX(ML) files in a chemistry table format. The ChemDraw libraries also allow the translation of some information that is added in form of common abbreviations that are added to the description of atoms. These abbreviations are the equivalent to molfile superatoms [31]. The summary of larger fragments in molecules is a common standard for example to represent protective groups attached to heteroatoms. For all those cases, which cannot be translated via the ChemDraw-integrated library, a library of 4300 superatoms consisting of a list of protective groups extracted from the well-known textbook “Greene's Protective Groups in Organic Synthesis” is applied [32]. The list is complemented by a manually collected and curated list of superatoms of the ChemScanner group and superatoms that are available by OpenBabel (see Fig. 2).

Textual information in the extracted schemes has to be split into several groups with the need to define different methods to re-use it. ChemDraw itself provides a Name = Struct [33, 34], an option which is used for the standard names and abbreviations inside CDX documents. Additionally to the Name = Struct method, a library of 6400 names given as text in form of trivial name or abbreviation or as the non-ambiguous formula is embedded to ChemScanner. The library consists of abbreviations and formula collected by the ChemScanner group as an internal library (approx. 1000 definitions) which were merged with names, abbreviations, and formula that were extracted from the Chemdner Corpus [19]. To this aim, the Chemdner corpus, an external library of 13 k abbreviations, 25 k trivial names and 12 k formula was extracted as published and was curated via an approval of the abbreviations and names with PubChem. Information that could be identified with PubChem was converted into SMILES and added to the ChemScanner library. For those items where no matching was found, manual curation allowed further identification of SMILES codes which were also added to ChemScanner. The remaining unsolved items were ignored. In the cases, where the ChemDraw library results in other SMILES translations than the ChemScanner library, ChemScanner obtains priority over the original ChemDraw suggestion.

All textually identified compounds are assigned to a specific role as reagent or solvent according to their matching with a predefined library of solvents and are stored as SMILES. In the last step, the textual information is also analyzed towards the presence of keywords indicating the availability of the parameters time, temperature and solvent. This information is separated from the further, not identified, text fragments (see Fig. 2).

**Fig. 3:**
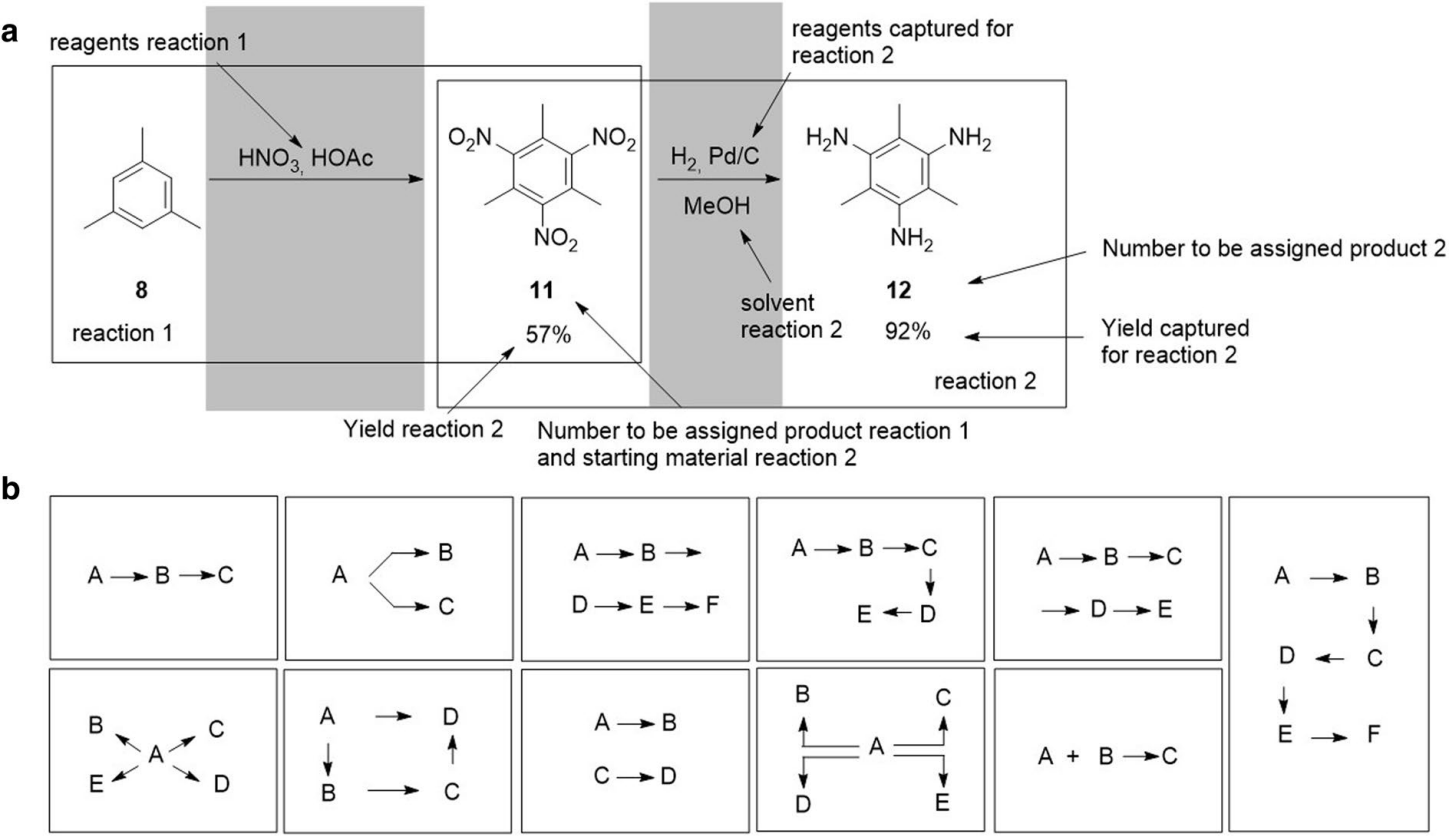
Explanation of the analysis mode for objects included in CDX and CDXML files: **a** correlation of geographic information and reaction role assignment; **b** schematic summary of some successfully assigned scenarios

Besides the described molecular and textual data, the geometry information of each object is also given within the CDX internal structure. Based on this geometry information, the CDX or CDXML scheme is analyzed concerning the position of every single object allowing the assignment to a special role as e.g. starting material, reactant, product, or solvent. Objects (of structural or textual nature) that are part of a certain area above or below the reaction arrow are determined to be reagents or solvents, while the solvents are extracted via an additional step that includes the comparison of the given SMILES string with a library of predefined solvents. Structures located left to the arrow are starting materials while all data right to the arrow is considered to be the product. This mechanism allows, after adaptions to the parsing of multi-arrow systems, the analysis of complex CDX(ML) schemes. Multistep reactions, for example, can be split into single step reactions which enables a standardized way for the representation of such processes in databases (Fig. 3a). As scientists use different ways to arrange their multistep reactions summarized in ChemDraw files, the ChemScanner has to deal with several challenging representations. A summary of the successfully implemented arrangement types is given schematically in Fig. 3b. Examples for the scope and limitations of the functionality are described in the Supporting Information (including so far unsolved presentation scenarios).

### Option 2: Processing of DOC or DOCX documents

Chemical procedures and project outcomes are often given not as CDX native but as DOC and DOCX documents as this offers the possibility to add more textual information. Technically, the DOCX^3^ file is a zip like archive consisting of an XML file and separated binaries including embedded CDX files. On the other hand, the DOC [35] file is a compound binary file (Microsoft Word binary file format) which can store other binaries (like CDX) within its internal structure. The ChemScanner induces a separation of the textual components of the DOC or DOCX document and an extraction of the embedded CDX binary files by following the specification of Perkin Elmer [36]. As soon as the embedded CDX binary files are isolated, they can be processed according to the procedure described above (“Option 1”).

### Option 3: Processing of XML files

The extraction of information from XML files was achieved for data exported from the Perkin Elmer ChemOffice ELN software. The feature is needed for ChemOffice users who want to re-use their previously collected information either for an import to another ELN or the generation of reactions or sample lists. For the extraction of the given data, the CDXML content of the XML file was separated and treated according to the CDXML files in “Option 1” section. Other information like the amount of chemicals participating in the reaction and data from the reaction table, the conditions, solvents and observations can be retrieved as well (see supporting information for a full list and mapping of the information). The extraction of the XML format offers additional valuable opportunities due to the availability of overlapping information from the extracted CDXML file and the reaction table. Information obtained by the CDXML file can be compared to the information of the reaction table by translation and comparison of both on the basis of the SMILES identifier. This procedure allows an approval of the extraction by means of identity of the compounds and completeness of the extracted structural and textual information.

### The ChemScanner UI and re-use of the extracted data

**Fig. 4:**
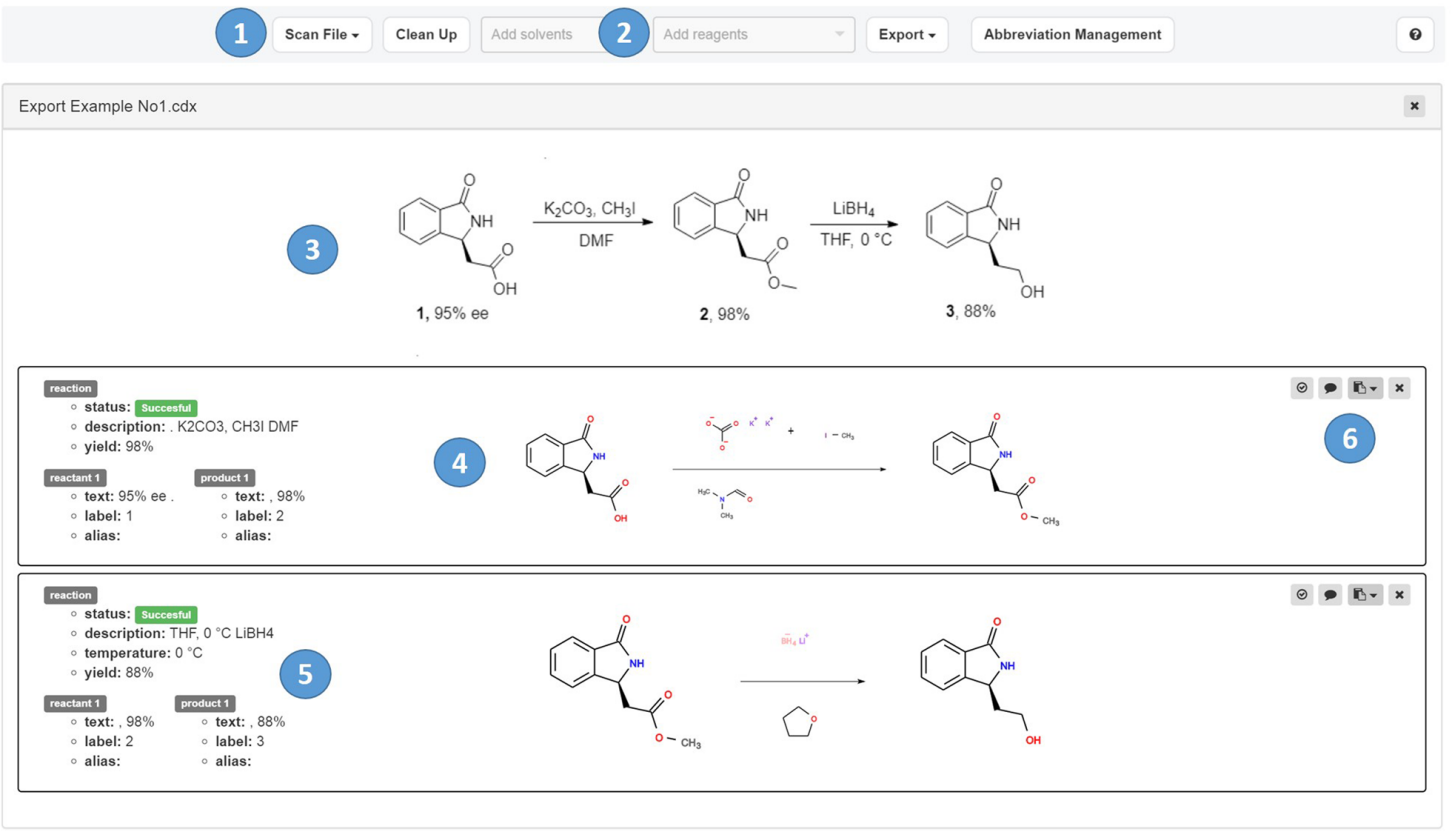
Screenshot of the ChemScanner UI after extraction of a two-step reaction and labeling of the most important features (size of the schemes was increased to improve the readability in the given view). Labels: 1, upload of documents or files via an easy drag and drop procedure or a selection of stored files; 2, Adding reagents or solvents via a dropdown list of 2000 entries; 3, preview of the original file as given by the upload in the web-application of Chemotion ELN (this add-on function needs a ChemDraw license but is not essential for the ChemScanner function); 4, visualization of extracted structures to verify the result; 5, representation of textual information in the scheme sorted according to the role of the identified items; 6, icons to access the functions “select reaction”, “add comment”, “copy reaction SMILES”, “copy molfiles” and “delete reaction”

The ChemScanner UI was built for managing the upload of different file types, the visualization and editing of the results, as well as the re-use and export of data. The user can either upload single or multiple files or can add files by the drag and drop routine from a personal storage. One can select between parsing of documents containing molecules or reactions. The results are displayed as images to allow proof-reading the conversion of the files for correctness and completeness. Being aware of the challenges of an extraction routine that has to deal with non-standardized input, especially because of the presence of additional textual information, we implemented options to add missing reagents and solvents and to comment on false structures or information manually. To this aim, a library of more than 2000 most common reagents and catalysts were collected and the contents can be added to a selected reaction or reaction group (see Fig. 4, label 2). The functionality of the ChemScanner UI is designed to fasten the approval or rejection of the processed data, and, if necessary, to correct quickly minor errors via this library. If the extraction fails completely or major errors are detected, the user is suggested to export the data and correct the wrong parts with a tool that allows structure editing (see the following chapter on export and re-use options). This procedure is facilitated by the option to add comments next to an extracted scheme which can be used within ChemScanner to indicate the errors or missing information (see Fig. 4, label 6). Additionally to the correction mode of the ChemScanner UI, a flagging of the reactions according to their success was incorporated: The ChemScanner automatically extracts indications on the positive or negative outcome of a reaction e.g. incorporated by a strike through an arrow as a signal for a failed reaction. Dashed arrows are interpreted as planned reactions. This allows a suggestion of whether the reaction was successful or not. This is an important feature as research plans or reports may contain negative results as well, which have to be indicated clearly during the extraction procedure (see Fig. 4, label 5).

**Fig. 5:**
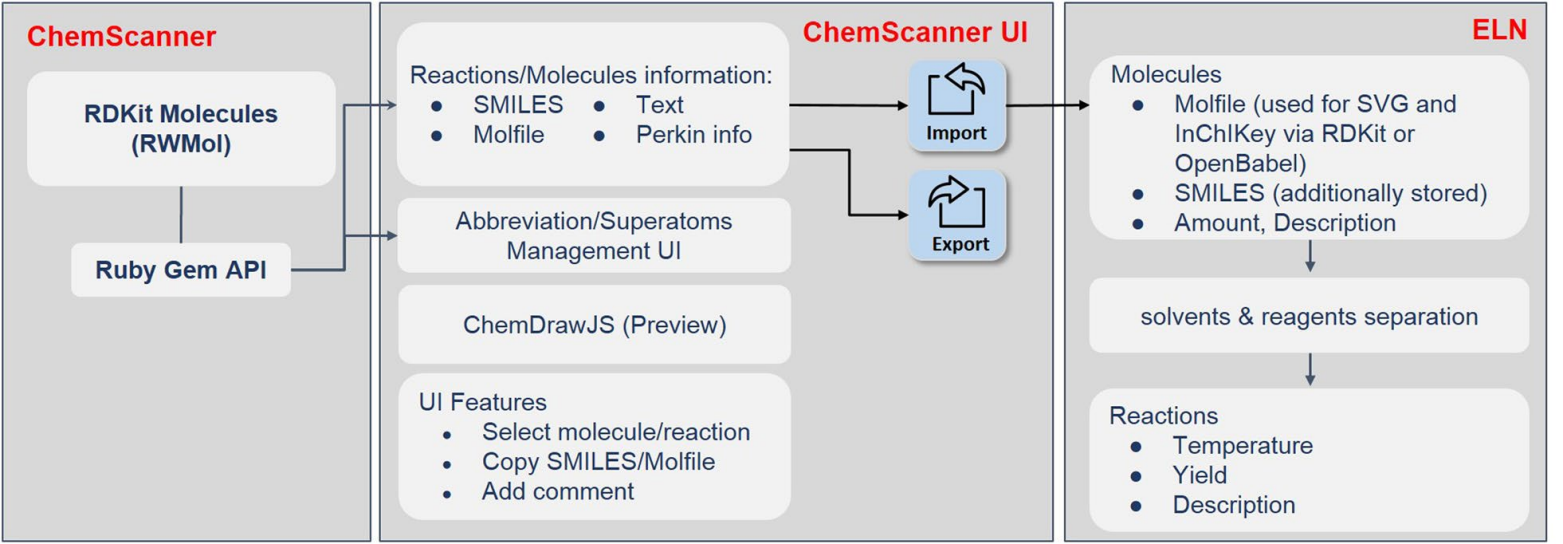
Main workflow describing the re-use of extracted information in the ELN. Dependencies of molecule identifiers and representation on the extracted information and its processing

To support further work with the data that can be extracted with ChemScanner and its re-use, the UI offers three options: one can (1) copy single structure information as reaction SMILES to the clipboard, (2) one can export the obtained and approved information to Excel or CML or (3) one can transfer the information directly to the Chemotion-ELN (see Additional file 1: Figure S1 as an example). The Chemotion ELN supports a facilitated editing and the availability of further chemoinformatic and management functions. The molecules are transferred to the ELN as Molfile and SMILES but Molfile as standard representation format has priority over the SMILES code with respect to the conversion of the molecule to an SVG and the generation of other identifiers via OpenBabel or RDKit (see Fig. 5). While the export functions are part of the gem, the transfer of data to the Chemotion ELN requires either the installation of the full Chemotion-ELN software (including ChemScanner) or the use of the demo version on https://www.chemotion.net.

### Discussion of the limitations of the current developments

At the current state of the developments of the ChemScanner, the software still has some limitations. The most important 5 limitations are given here to give a transparent description of the potential developments.

1. At the moment, ChemScanner recognizes R-groups as undefined residues and inserts an asterisk (*) in the position of the undefined residue. If the position R is well-defined (e.g. R = Me), ChemScanner is able to substitute the R, if it is not well-defined (e.g. R = 4-F-C6H4), ChemScanner will lose the information (see example Additional file 1: Figure S4-1).
2. ChemScanner extracts information on coordinative bonds but the information cannot be used for a SMILES export because coordinative bonds do not have a representation in SMILES. The current procedure deals with a Molfile representation to include the coordinative bond information (as bond type 9). Users who export the extracted data only with SMILES have to consider this point (see Additional file 1: Figure S4-2).
3. In special cases where stereochemistry is not indicated by wedged or hashed wedged bonds, ChemScanner does not convert information on the stereochemistry of the drawn structures to the SMILES. This may be the case for example for carbohydrates (see Additional file 1: Figure S4-3). The SMILES is given in those cases with undefined stereocenters. This limitation can be circumvented by import of the ChemScanner results (as Molfile) to the Chemotion ELN (e.g. web application).
4. Some very complex examples for arrangements of multistep reactions are not extracted correctly yet (see example in Additional file 1: Figure S4-4).
5. Some translation errors are already inserted at the level of the native ChemDraw document and we were not able to check for all possible errors (see Additional file 1: Figure S4-5).

The authors will continue their work on the improvement of ChemScanner and hope to be able to solve the remaining challenges during the next months. For additionally discovered mistakes, the ChemScanner group will be glad to add further corrections to the source code.

## Conclusion

We describe the development and application of ChemScanner, a software that extracts chemistry information from native CDX and CDXML files or DOC, DOCX or XML documents that contain CDX or CDXML files. The extracted information is processed to reaction and molecule entities, as well as additional text and values and can be accessed via the ChemScanner UI. ChemScanner supports the export to Excel, to CML, the import of the extracted data directly to the Open Source ELN Chemotion or the use via “copy and paste” of selected information. The ChemScanner was developed with the use of several helpers like RDKit (OpenBabel could be used alternatively) and PubChem and was built on previous work like Chemdner. Additionally, many improvements—in particular with respect to the available libraries—have been added during the project phase. ChemScanner was adapted to many typical challenges that have to be solved to extract common chemical presentations. Examples are the implementation of diverse possible reaction scheme arrangement styles for multistep reactions, the detection of compound labels, reaction conditions and other information or the extraction of indications on the success of a reaction. Several additional helpers that support the adaption, correction and correct export or transfer of the extracted information were implemented. The developed software can be used for the extraction of basic chemical information included in many chemical drawings. Anyhow, the software still has limitations with respect to certain applications. These limitations are discussed briefly. We hope that ChemScanner will support to re-use chemical information embedded to documents or native ChemDraw files. The installed web-service can be used for single extraction requests but also for an augmentation of data by a step by step extraction of several documents and the storage of the results.

### Availability and requirements

- Project name: ChemScanner.
- Project homepage: https://www.eln.chemotion.net.
- Operating system(s): Platform independent access, developed/tested on Linux and Mac, deployed on Linux.
- Library requirements (for developer): Ruby version 2.2 or later, 7z [37] and a customized version of RDKit [29].
- Other requirements: Modern internet browser supporting HTML5 and JavaScript.
- Recommended browsers: Chrome, Firefox (IE not supported).
- Programming language: Ruby.
- Source Code: https://github.com/complat/chem_scanner.
- Licence: AGPLv3.

## Supplementary information

**Additional file 1.** The additional file provides supplemental information describing details of the software and programming aspects. Examples that illustrate the scope and limitations of the extraction of challenging CDX and CDXML files are given

## Data Availability

Additional file 1 covers technical aspects and details of the software and programming e.g. the installation requirements for the full Chemotion-ELN software including the ChemScanner and the basic use of ChemScanner. It contains a table to describe the mapping of the XML information to the ChemScanner UI and the ELN import. The Supporting Information contains further examples illustrating the scope and limitations of the CDX and CDXML functionality for extraction of challenging CDX and CDXML files including multistep procedures.

## Abbreviations

ELN: electronic Laboratory Notebook
UI: user interface
XML: extensible Markup Language
CDX: chemDraw Exchange

## References

[1] https://www.nature.com/articles/sdata201618. Accessed 6 Sept 2018

[2] https://www.rd-alliance.org/about-rda. Accessed 6 Sept 2018

[3] ChemDraw, a product of PerkinElmer. https://www.cambridgesoft.com/software/overview.aspx

[4] Li Z, Wan H, Shi Y, Ouyang P (2004). Personal experience with four kinds of chemical structure drawing software: review on ChemDraw, ChemWindow, ISIS/Draw, and ChemSketch. J Chem Info Comp Sci.

[5] https://chemaxon.com/products/marvin. Accessed 6 Sept 2018

[6] Ketcher. https://lifescience.opensource.epam.com/ketcher/#overview. Accessed 6 Sept 2018

[7] Kotov S, Tremouilhac P, Jung N, Bräse S (2018). Chemotion-ELN part 2: adaption of an embedded Ketcher editor to advanced research applications. J Cheminform.

[8] Krause S, Willighagen E, Steinbeck C (2000). JChemPaint—using the collaborative forces of the: 1 to develop a free editor for 2D chemical structures. Molecules.

[9] Bienfait B, Ertl P (2013). JSME: a free molecule editor in JavaScript. J Cheminform.

[10] Southan C, Stracz A (2013). Extracting and connecting chemical structures from text sources using chemicalize.org. J Cheminform.

[11] https://chemaxon.com/products/chemical-data-extraction. Accessed 6 Sept 2018

[12] https://cactus.nci.nih.gov/osra/. Accessed 6 Sept 2018

[13] Filippov IV, Nicklaus MC (2009). Optical structure recognition software to recover chemical information: OSRA, an open source solution. J Chem Inform Model.

[14] Krallinger M, Rabal O, Lourenço A, Oyarzabal J, Valencia A (2017). Information retrieval and text mining technologies for chemistry. Chem Rev.

[15] Corbett P, Murray-Rust P (2006). High-throughput identification of chemistry in life science texts. Lect Notes Comput Sci.

[16] Lowe DM, Corbett PT, Murray-Rust P, Glen RC (2011). Chemical name to structure: OPSIN, an open source solution. J Chem Inform Model.

[17] Lowe DM (2012) Extraction of chemical structures and reactions from the literature. Ph.D. Thesis, University of Cambridge. DSpace.

[18] Lowe DM, Murray-Rust P, Glen RC (2013) OPSIN: taming the jungle of IUPAC chemical nomenclature. In: 6th joint sheffield conference on chemoinformatics. https://app.box.com/s/8u4vewvfu3sol3axli3r

[19] Krallinger M, Leitner F, Rabal O, Vazquez M, Oyarzabal J, Valencia A (2015). CHEMDNER: the drugs and chemical names extraction challenge. J Cheminform.

[20] Hawizy L, Jessop DM, Adams N, Murray-Rust P (2011). ChemicalTagger: a tool for semantic text-mining in chemistry. J Cheminform.

[21] Jessop DM, Adams SE, Willighagen EL, Hawizy L, Murray-Rust P (2011). OSCAR4: a flexible architecture for chemical text-mining. J Cheminform.

[22] Papadatos G, Davies M, Dedman N, Chambers J, Gaulton A, Siddle J, Koks R (2016). SureChEMBL: a large-scale, chemically annotated patent document database. Nucleic Acids Res.

[23] Lowe DM (2014) Patent reaction extraction: downloads. https://bitbucket.org/dan2097/patent-reaction-extraction/downloads

[24] O'Boyle NM, Banck M, James CA (2011). Open Babel: an open chemical toolbox. J Cheminform.

[25] RDKit: open-source cheminformatics. http://www.rdkit.org. Accessed 8 Dec 2019

[26] Tremouilhac P, Nguyen A, Huang Y-C, Kotov S, Lütjohann DS, Hübsch F, Jung N, Bräse S (2017). Chemotion ELN: an Open Source electronic lab notebook for chemists in academia. J Cheminform.

[27] https://github.com/complat/chem_scanner. Accessed 26 Nov 2019

[28] https://www.perkinelmer.com/product/e-notebook-enotebook. Accessed 6 Sept 2018

[29] https://github.com/CamAnNguyen/rdkit_chem. Accessed 30 July 2019

[30] Icons made by Freepik. https://www.flaticon.com

[31] Gushurst AJ, Nourse JG, Hounshell WD, Leland BA, Raich DG (1991). The substance module: the representation, storage, and searching of complex structures. J Chem Inform Model.

[32] Wuts PGM, Greene TW (2006) Greene's protective groups in organic synthesis, 4th edition. Wiley. ISBN: 978-0-470-05348-5

[33] Brecher J (1999). Name=Struct: a practical approach to the sorry state of real-life chemical nomenclature. J Chem Inform Comput Sci.

[34] https://www.cambridgesoft.com/support/DesktopSupport/Documentation/N2S/. Accessed 6 Sept 2018

[35] https://msdn.microsoft.com/en-us/library/office/cc313153(v=office.12).aspx. Accessed 6 Sept 2018

[36] https://www.cambridgesoft.com/services/documentation/sdk/chemdraw/cdx/index.htm. Accessed 6 Sept 2018

[37] https://www.7-zip.org/. Accessed 6 Sept 2018

