# Supplementary material for: ChemScanner: extraction and re-use(ability) of chemical information from common scientific documents containing ChemDraw files
Source: J Cheminform. 2019 Dec 11;11:77. doi: 10.1186/s13321-019-0400-5 (PMC6907231; doi:10.1186/s13321-019-0400-5)
Supplement: Supplementary file 1 — Additional file 1. The additional file provides supplemental information describing details of the software and programming aspects. Examples that illustrate the scope and limitations of the extraction of challenging CDX and CDXML files are given [file 13321_2019_400_MOESM1_ESM.pdf]

ChemScanner: Extraction and re-use(ability) of chemical information from common scientific documents containing ChemDraw files

An Nguyen[a], *Yu-Chieh Huang*[a], *Pierre Tremouilhac*[a], *Nicole Jung** [a,b], *Stefan Bräse** [a,b]

Email: an.nguyen@kit.com; yu-chieh.huang@kit.edu; pierre.tremouilhac@kit.edu; nicole.jung@kit.edu; stefan.braese@kit.edu

[a]Institute of Toxicology and Genetics, Karlsruhe Institute of Technology, Hermann-von-Helmholtz-Platz 1, 76344 Eggenstein-Leopoldshafen, Germany; [b]Institute of Organic Chemistry, Karlsruhe Institute of Technology, Fritz-Haber-Weg 6, 76131 Karlsruhe, Germany.

Contents

1. Technical Aspects 2

2. Table for mapping details XML-ChemScanner-ELN import.. 3

3. Import of extracted reactions to chemotion-ELN 4

3.1 Import of reactions obtained from CDX 4

3.2 Import of reactions obtained from CDXML documents (PerkinElmer E-Notebook)........ 7

4. Examples indicating scope and limitations of CDX/CDXML interpretation 9

1. Technical Aspects

ChemScanner is a Ruby gem developed for Ruby 2.3.3 or later version. ChemScanner uses 7zip for uncompressing Office documents, and a Ruby-binding RDKit version as chemistry library for molecule processing. ChemScanner UI is part of Chemotion ELN (version > 0.5.0). The source code of Chemotion_ELN is available at: https://github.com/ComPlat/chemotion_ELN and https://git.scc.kit.edu/ComPlat/chemotion_eln_server

The Chemscanner UI can be tested at: https://eln.chemotion.net/chemscanner. ChemScanner source code is available at https://github.com/complat/chem_scanner). The repository includes instructions, API and license details.

Installation notes for Chemotion ELN server can be found at: https://git.scc.kit.edu/ComPlat/chemotion_eln_server/wikis/home

A Virtual Machine (VM) template with preinstalled Chemotion ELN for production or development environments can be accessed at: https://git.scc.kit.edu/ComPlat/chemotion_eln_server/wikis/vm-template. A VM template with preinstalled ChemScanner can also be provided on request.

2. Table for mapping details XML-ChemScanner-ELN import

Perkin Elmer E-Notebook	Chemotion Electronic Lab Notebook (ELN)
**Reaction**	**Reaction**
Chemical Structure (the CDXML scheme)	Scheme
Temperature	Temperature
Reaction Molarity	Description
Pressure	Description
Preparation	Description
**Reactants**	**Starting Materials/Reactants/Reagents**
• Starting Materials/Reactants/Reagents in ELN are given in “Reactants” list in E-Notebook<br>• Samples are determined via ID and/or SMILES from the CDXML scheme<br>• Importing will attempt to translate samples whose SMILES are not given based on the predefined list. If failed, these samples information will be import to reaction’s description	
Name	Name (and description)
Molarity	Molarity
Density	Density
Moles	Amount (mol)
Molecular Formula	Ignored (Derived from structure)
Molecular Weight	Ignored (Derived from structure)
Equivalents	Ignored (Derived reaction’s amount)
Sample Mass	Ignored (Derived from structure and Moles)
Volume	Ignored (Derived from Moles and Molarity/Density)
Limiting?	Ignored
Reaction Label	Ignored
% by Weight	Ignored
Formula Mass	Ignored
Reactant Mass	Ignored
Barcode	Ignored
Compound ID	Ignored
CAS	Ignored
Supplier	Ignored
Reg Number	Ignored
Batch Number	Ignored
Sample ID	Ignored
**Products**	**Products**
Name	Name (and description)
Actual Moles	Amount
Reaction Label	Description
Molecular Formula	Ignored (Derived from structure)
Molecular Weight	Ignored (Derived from structure)
Actual Mass	Ignored (Derived from structure and Moles)
% Yield	Ignored (Derived reaction’s amount)
% Purity	Ignored (Derived reaction’s amount)
Equivalents	Ignored (Derived reaction’s amount)
Theoretical Moles	Ignored
Theoretical Mass	Ignored
Formula Mass	Ignored
Product ID	Ignored

3. Import of extracted reactions to chemotion-ELN

3.1 Import of reactions obtained from CDX

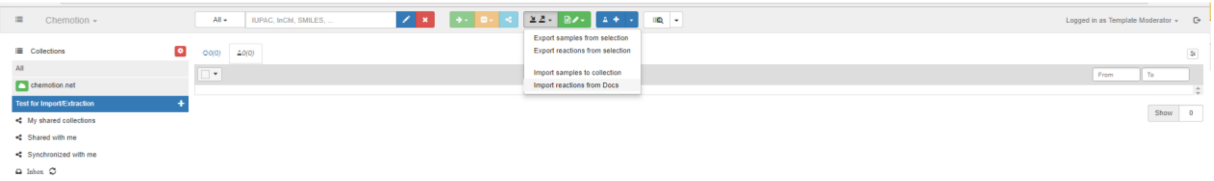

Figure S3-1: View of ELN user interface: Selection of action: “import reaction from docs”

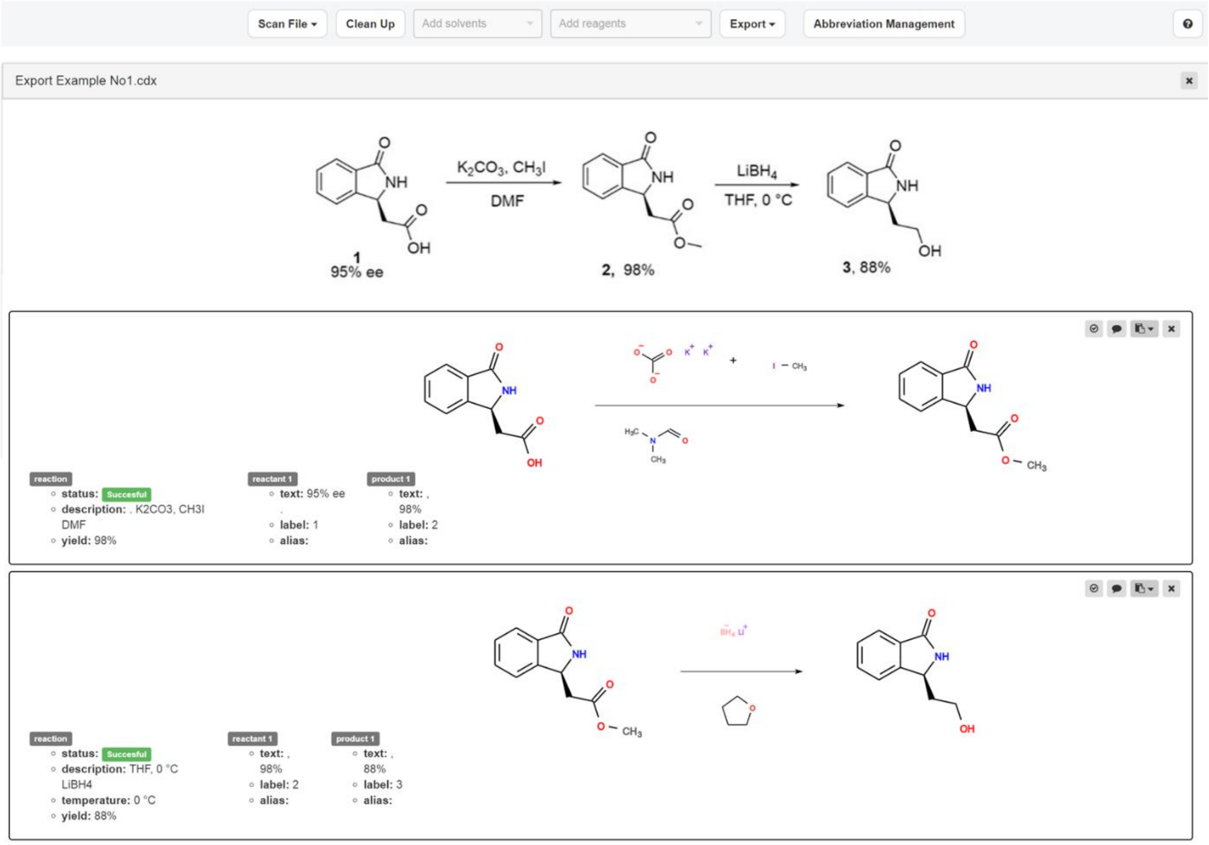

Figure S3-2: View in ChemScanner UI after selection of a CDX file: extraction of contents and visibility (size of the schemes was increased to improve the readability in the given view).

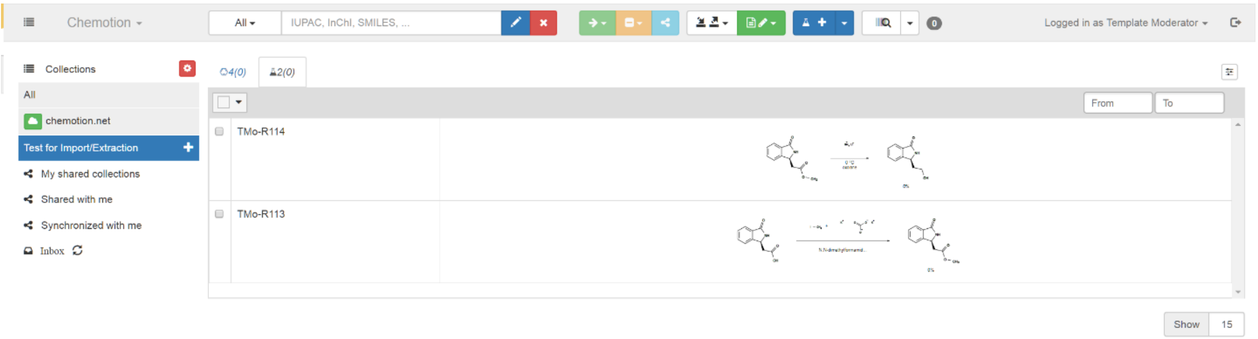

Figure S3-3: View in ELN after import of extracted reactions, amount of imported reactions: 2.

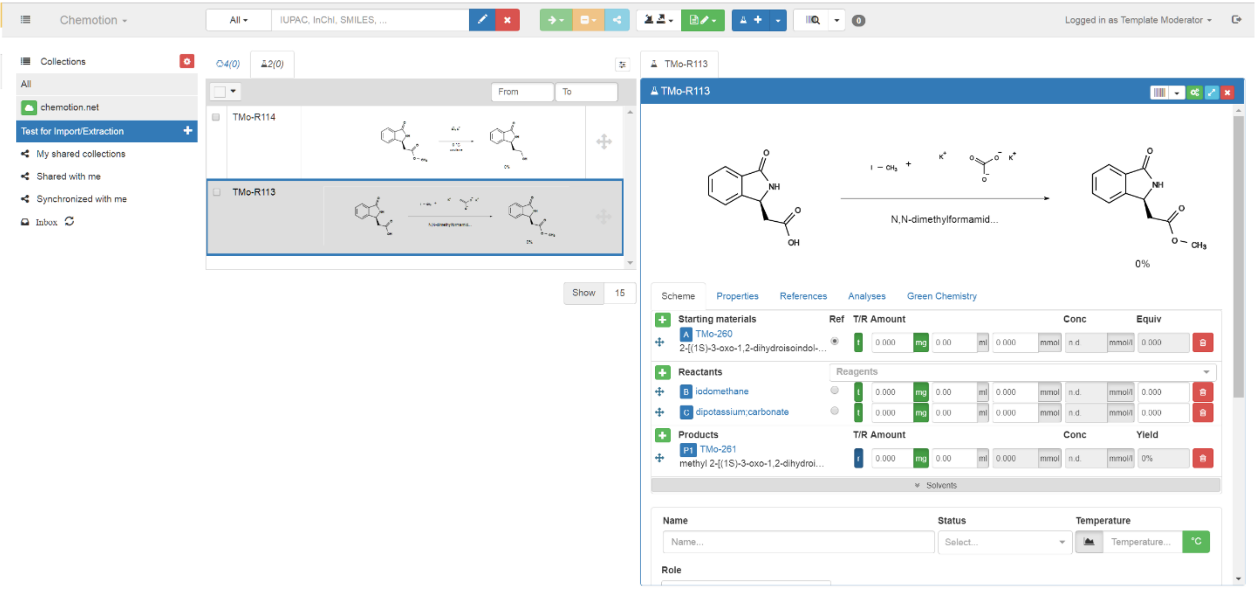

Figure S3-4: Detailed view of an imported reaction: most information fields for the registered compounds are empty in the details tab due to missing information in the CDX file (only ChemDraw scheme without additional information was given).

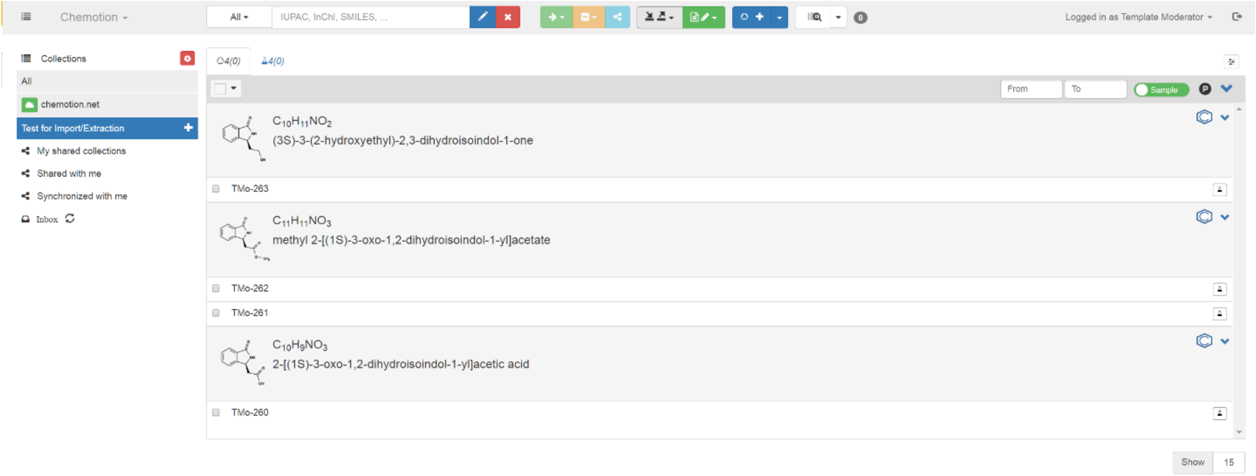

Figure S3-5: View in the ELN after import of reactions: listing of contained samples and molecules. Registered molecules: 3; registered samples: 4 (one molecule was registered as starting material and product).

3.2 Import of reactions obtained from CDXML documents (PerkinElmer E-Notebook)

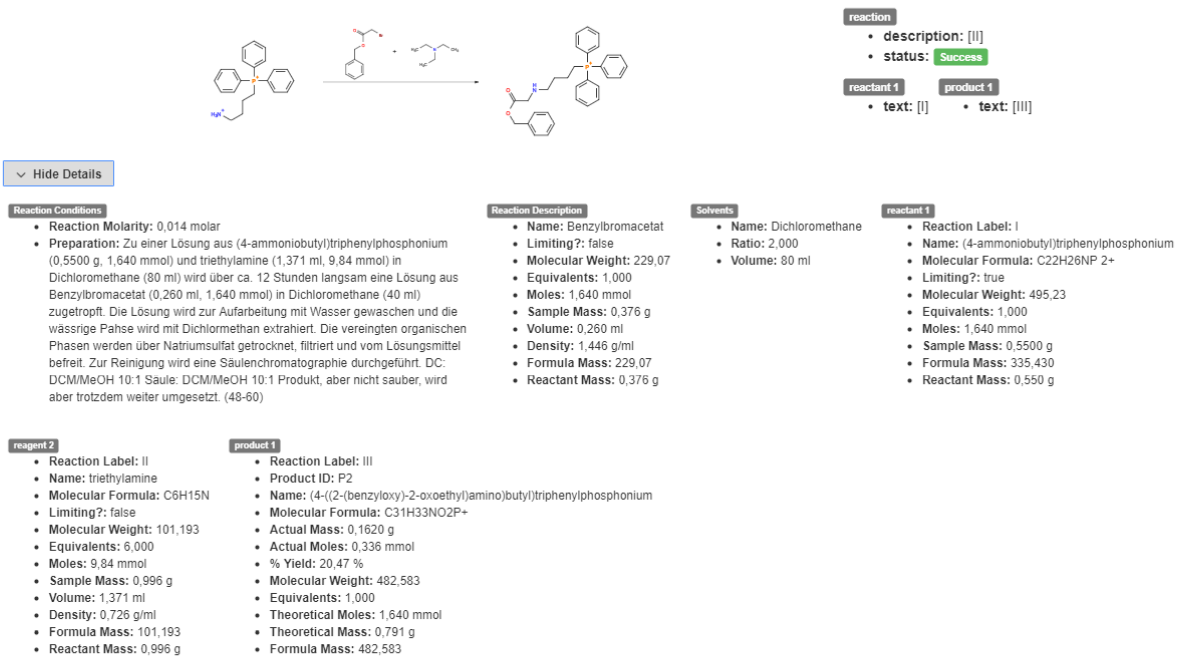

Figure S3-6: View in ChemScanner UI after extraction of a reaction from PerkinElmer E-Notebook, (only panel view given to improve the readability).

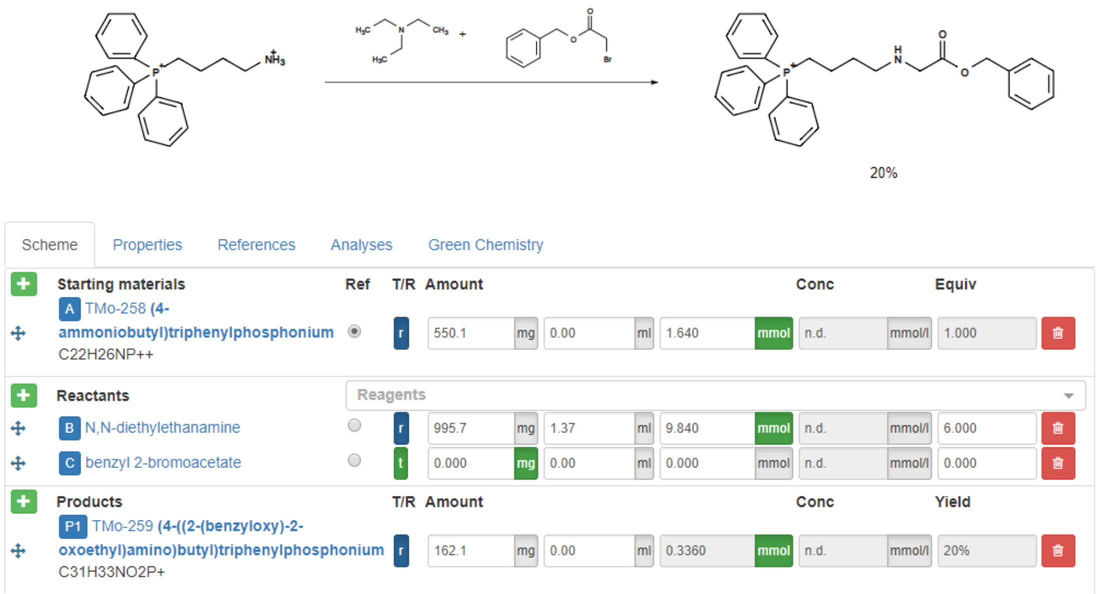

Description

solvents:

+ **ID:**
+ **parentID:**
+ **Name:** Dichloromethane
+ **Ratio:** 1,000
+ **Volume:** 40 ml

[II]

+ **Reaction Molarity:** 0,014 molar
+ **Preparation:** Zu einer Lösung aus (4-ammoniobutyl)triphenylphosphonium (0,5500 g, 1,640 mmol) und triethylamine (1,371 ml, 9,84 mmol) in

Description

+ **Reaction Molarity:** 0,014 molar
+ **Preparation:** Zu einer Lösung aus (4-ammoniobutyl)triphenylphosphonium (0,5500 g, 1,640 mmol) und triethylamine (1,371 ml, 9,84 mmol) in Dichloromethane (80 ml) wird über ca. 12 Stunden langsam eine Lösung aus Benzylbromacetat (0,260 ml, 1,640 mmol) in Dichloromethane (40 ml) zugetropft. Die Lösung wird zur Aufarbeitung mit Wasser gewaschen und die wässrige Pahse wird mit Dichlormethan extrahiert. Die vereingten organischen Phasen werden über Natriumsulfat getrocknet, filtriert und vom Lösungsmittel befreit.
Zur Reinigung wird eine Säulenchromatographie durchgeführt.

DC: DCM/MeOH 10:1
Säule: DCM/MeOH 10:1

Produkt, aber nicht sauber, wird aber trotzdem weiter umgesetzt.
(48-60)

Figure S3-7: Imported reaction from S3-6 into Chemotion ELN.

4. Examples indicating scope and limitations of CDX/CDXML interpretation

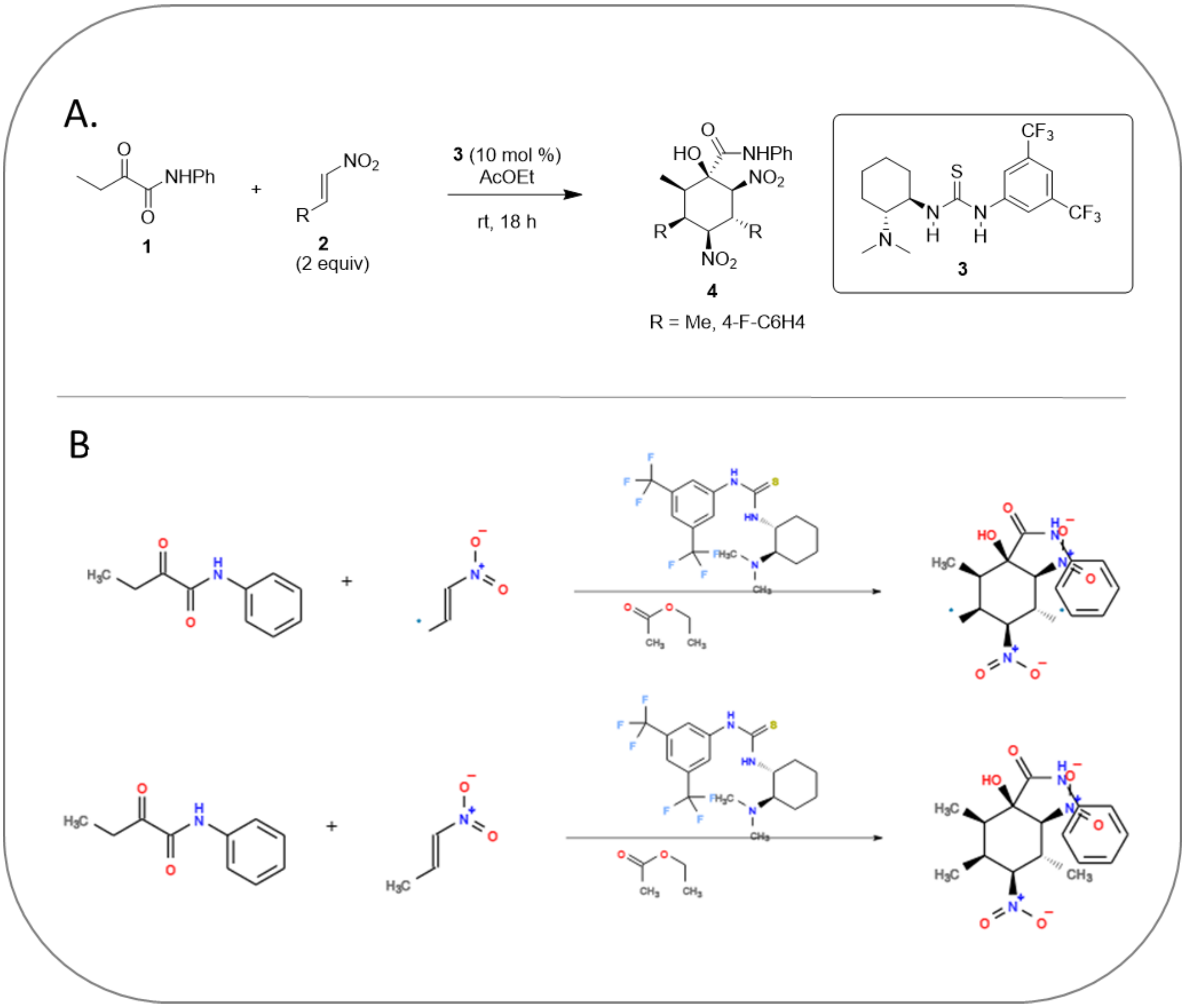

Figure S4-1: Extraction of structures containing R-groups, asterisk (*) indication in the position of the undefined residue.

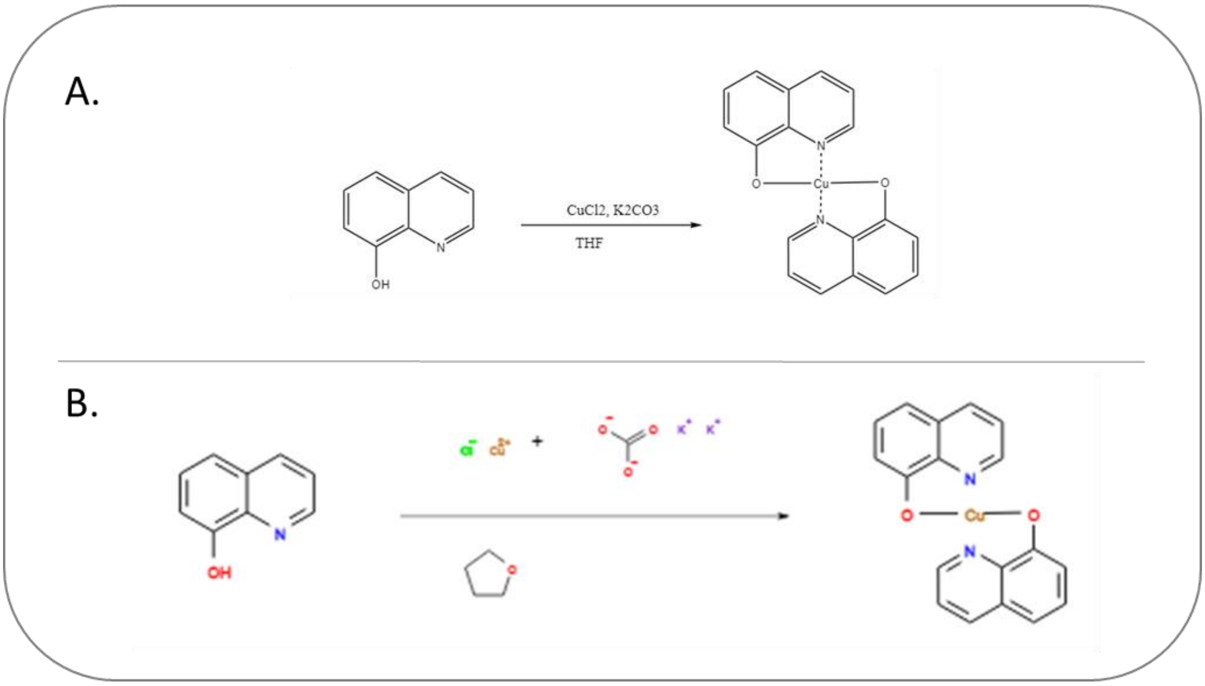

Difference between representation in SMILES and Molfile:

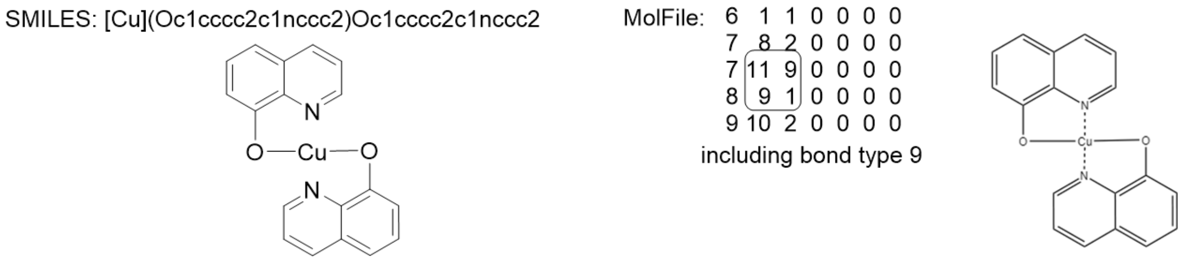

Figure 4-2: Extraction of compounds containing coordinative bonds and differences between the representation of the structure as SMILES or Molfile: While a structure extraction and re-use of the information as SMILES won't result in description as coordinative bond, a representation in Molfile and the use of the Molfile offers the representation including coordinative bonds (due to bond type 9).

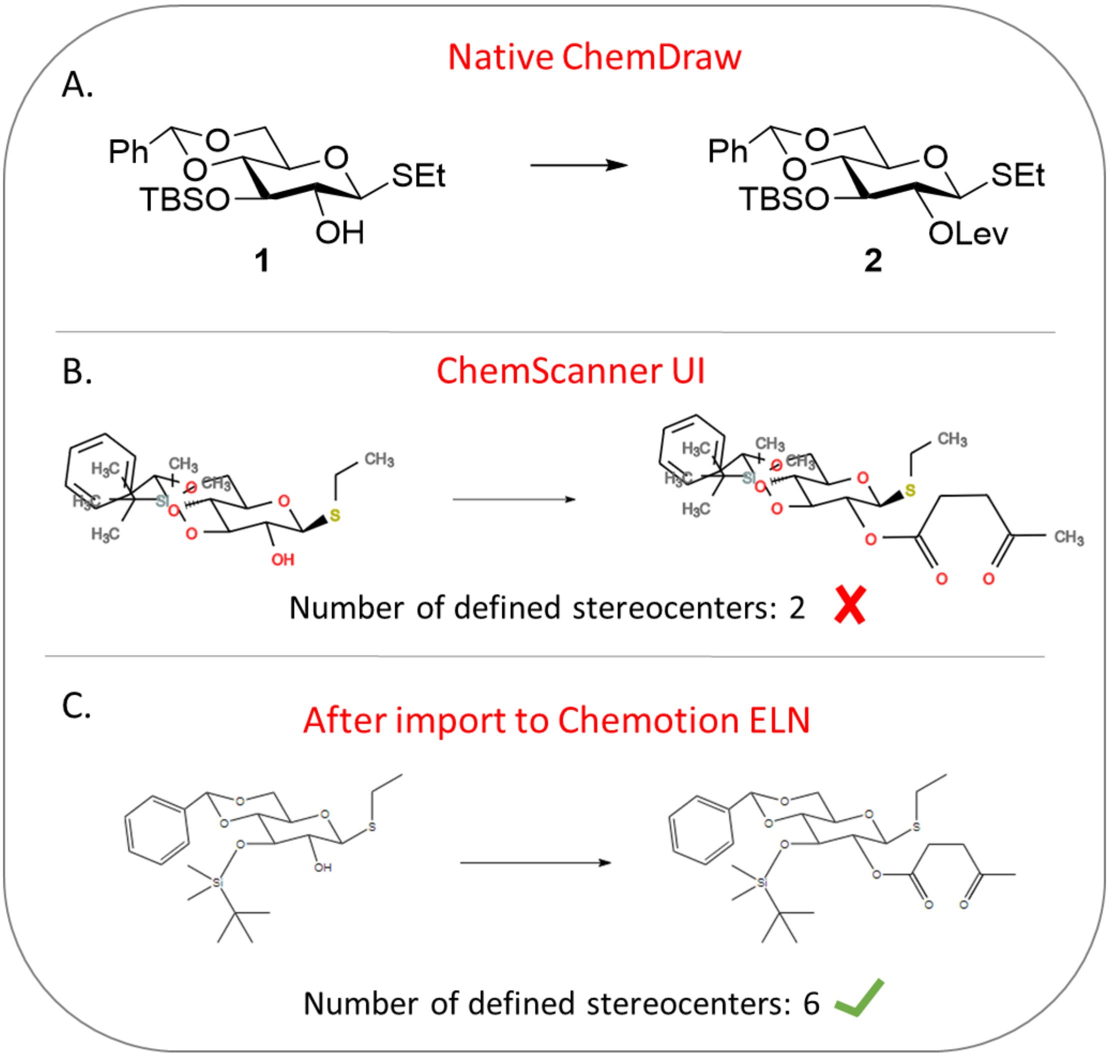

Figure S4-3: Lost information on stereochemistry in case of carbohydrates during the conversion of the ChemDraw file (scheme A) to the extracted structure information (scheme B). Scheme C describes to outcome and restoration of information via the import of the information to the Chemotion ELN (web-application) which includes a ChemDrawJS editor.

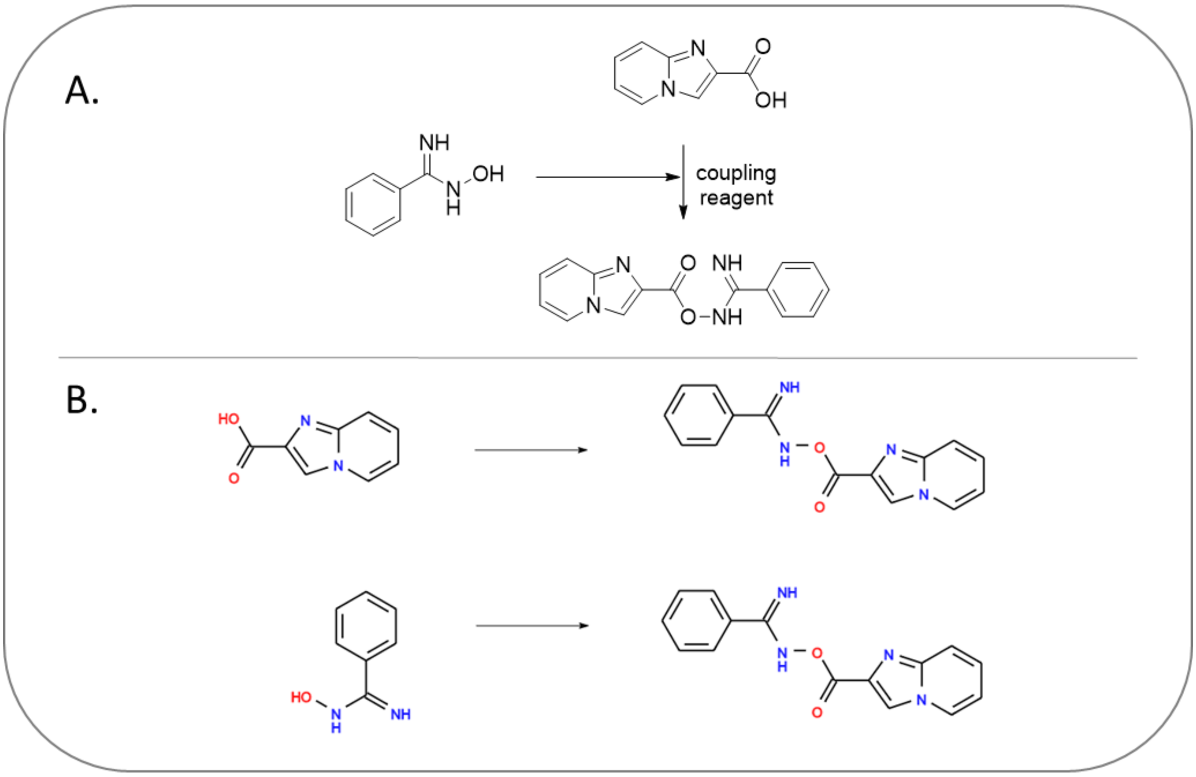

Figure S4-4: Multistep reactions that are not extracted correctly.

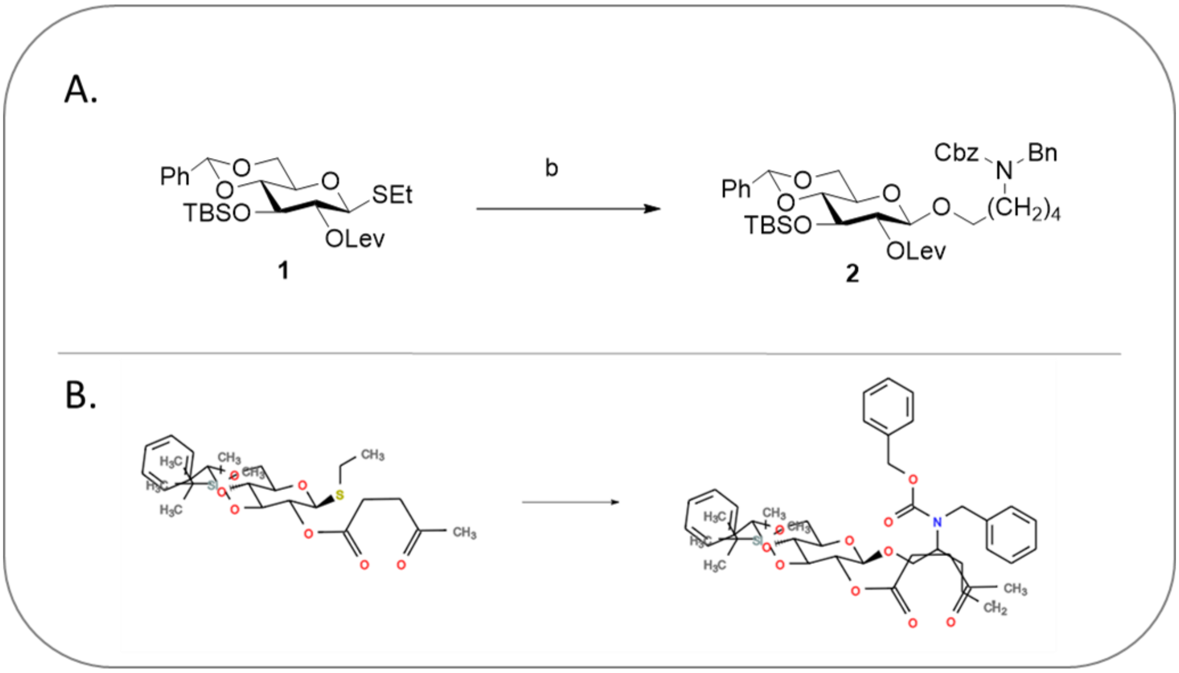

Figure S4-5: Translation errors caused by ChemDraw-internal interpretation.
